# Hepatic Fascioliasis: A Rare Cause of Portal Vein Thrombosis

**DOI:** 10.7759/cureus.103258

**Published:** 2026-02-09

**Authors:** Daniela Duarte, António Moreno Marques, Inês Pintado Maury, Alexandra Vaz

**Affiliations:** 1 Department of Internal Medicine, Unidade Local de Saúde de Viseu Dão-Lafões, Viseu, PRT; 2 Department of Infectious Diseases, Centro Hospitalar De Lisboa Norte, Lisbon, PRT

**Keywords:** eosinophilia, fasciola hepatica, hepatic fascioliasis, liver abscess, parasitic infection, portal vein thrombosis

## Abstract

Fascioliasis is a zoonosis caused by the trematodes *Fasciola hepatica* or *Fasciola gigantica*, acquired through ingestion of contaminated water or aquatic plants. Clinically, it presents with an acute phase characterized by fever, abdominal pain, and hepatomegaly, and a chronic phase, which is usually asymptomatic. Portal vein thrombosis (PVT) is a rare complication, not previously described among the approximately 30 cases of fascioliasis reported in Portugal. We describe a 48-year-old man who had been on a recent trip to Cape Verde and consumed unpackaged watercress. He was admitted with fever and right upper quadrant pain; laboratory results showed eosinophilia and recurrence of a hepatic abscess associated with PVT. Positive *Fasciola hepatica* serology and immunoblot confirmed the diagnosis. Treatment with triclabendazole led to a significant reduction of the abscess after three months. Fascioliasis should be considered in the differential diagnosis of hepatic abscesses in patients with epidemiological risk factors and poor response to conventional antibiotic therapy, to prevent possible complications.

## Introduction

Fascioliasis is a zoonotic disease caused by the trematodes *Fasciola hepatica* or *Fasciola gigantica*. The World Health Organization (WHO) classifies it as a neglected tropical disease, with worldwide distribution in more than 80 countries across all continents. Approximately 2.6 million people are infected, with an estimated global prevalence of 4.5%. The most affected regions are South America and Africa [1,2].

The scarcity of consistent epidemiological data, resulting from the lack of mandatory disease notification in Portugal, makes it difficult to estimate its prevalence in the country. According to the literature, 33 cases of fascioliasis have been reported in Portugal since 1984, 14 of which occurred in the last 25 years. Most cases are of autochthonous origin and are associated with the consumption of raw watercress or spring water in the Minho, Alto Douro, and Beira Interior regions [3-10].

Infection occurs through ingestion of water or aquatic plants, such as watercress, contaminated with encysted larvae (metacercariae), which are excreted in the feces of herbivorous animals, mainly sheep and cattle, the definitive hosts [11].

Clinical manifestations are divided into two phases. The acute or hepatic invasion phase lasts from two weeks to four months and is classically characterized by the triad of fever, epigastric or right upper quadrant pain, and hepatomegaly. Nonspecific symptoms such as myalgia, arthralgia, anorexia, weight loss, allergic manifestations (pruritus and urticaria), and the presence of hepatic abscess may also occur. The chronic or biliary invasion phase ranges from three months to 10 or more years. Most cases are asymptomatic. When symptoms are present, they may include recurrent cholangitis, gallstone disease, cholecystitis, obstructive jaundice, and pancreatitis [12].

Portal vein thrombosis (PVT) is a rarely described complication of fascioliasis. Among the approximately 30 cases reported in Portugal, none described the occurrence of PVT [3-10].

This case aims to raise awareness of a cause of hepatic abscess that has become less frequent due to improvements in hygienic and sanitary conditions but remains present and should still be considered, particularly in an era of globalization, to highlight a rare complication of fascioliasis.

## Case presentation

A 48-year-old man, previously healthy and not taking any regular medication, presented with right upper quadrant pain and fever. The patient reported a recent hospitalization for a right hepatic lobe abscess, during which he received empirical antibiotic therapy with ceftriaxone and metronidazole and underwent ultrasound-guided drainage, yielding dark serohematic fluid.

Microbiological studies were negative, including blood cultures, stool parasitological examination, and bacteriological analysis of the drained material. He was discharged after clinical and laboratory improvement on cefuroxime and metronidazole to complete a total treatment duration of four to six weeks, with follow-up in the Infectious Diseases outpatient clinic. From an epidemiological standpoint, a trip to Cape Verde six months earlier was noted, during which he had consumed non-bottled water and frequently ingested raw, unpackaged watercress.

One month later, the patient returned with persistent right upper quadrant pain and fever, with worsening laboratory results, including elevated inflammatory markers and a cholestatic pattern of liver injury. A follow-up abdominal ultrasound revealed a recurrence of the hepatic abscess and thrombosis of a segmental branch of the portal vein. Physical examination was unremarkable except for pain on deep palpation of the right upper quadrant.

Laboratory evaluation showed elevated inflammatory markers (C-reactive protein 6.46 mg/dL) and a cholestatic pattern of liver injury, with increased alkaline phosphatase (236 U/L) and Gamma-glutamyl transferase (GGT; 168 U/L), as well as mildly elevated transaminases (aspartate aminotransferase /alanine aminotransferase (AST/ALT) 114/95 U/L). Notably, eosinophilia was present (0.87 × 10⁹/L) (Table 1).

**Table 1:** Biochemistry workup performed at admission and after Fasciola hepatica treatment AST: aspartate aminotransferase, ALT: alanine aminotransferase, GGT: Gamma-glutamyl transferase, INR: International Normalized Ratio.

Laboratory test		Admission	Post-treatment	Normal ranges
White blood cells	x10^9^/L	5.60	7.0	4.0-11.0
Neutrophils	x10^9^/L	2.23	4.42	1.9-7.5
Eosinophils	x10^9^/L	0.87	0.08	0-0.5
Hemoglobin	g/dL	13.8	14.7	13-17.5
Platelets	x10^9^/L	239	204	150-450
INR		1.02	0.98	0.8-1.2
Blood urea	mg/dL	15	49	16-49
Serum creatinine	mg/dL	0.76	1.07	0.7-1.2
Serum sodium	mEq/L	139	139	135-145
Serum potassium	mEq/L	3.6	4.4	3.5-5.1
Alkaline phosphatase	UI/L	236	84	40-130
GGT	UI/L	168	28	0-60
ALT/AST	UI/L	114/95	46/31	0-40
Total bilirrubin	UI/L	0.43	0.67	<1.2
Procalcitonin	ng/mL	0.17	<0.02	<0.5
C-reactive protein	mg/dL	6.46	0.08	<0.5

As a part of the etiological workup, serological tests were requested for *Entamoeba histolytica, Toxocara, Schistosoma, Fasciola, Brucella, Coxiella burnetii, Bartonella henselae, Leishmania, *and *Toxoplasma gondii*. Additionally, interferon-gamma release assay (IGRA) and Venereal Disease Research Laboratory (VDRL) testing were performed.

**Figure 1:**
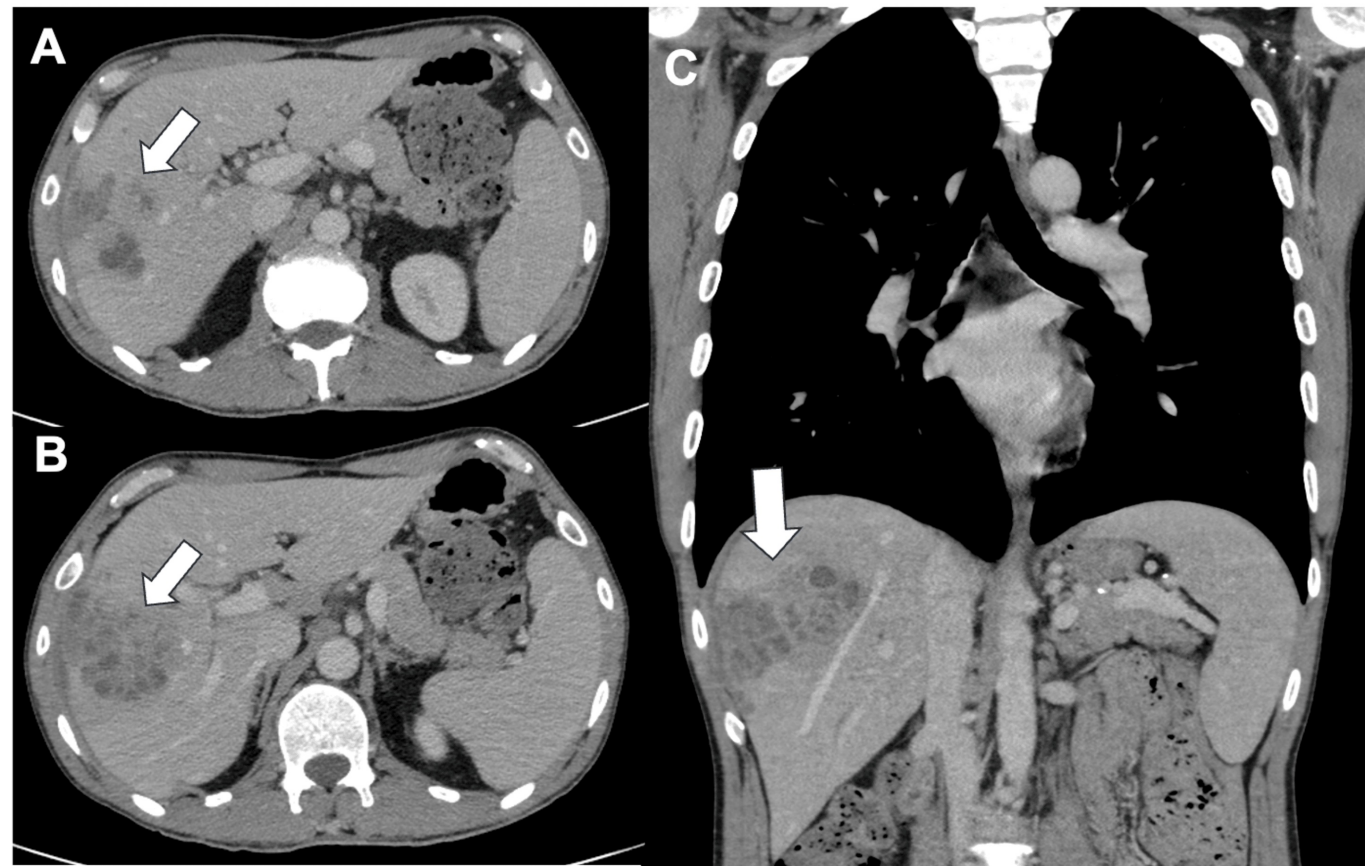
Abdominopelvic CT images A and B) Abdominal computed tomography (CT) axial images showing a heterogeneous hepatic collection with confluent microcystic areas and irregular margins (arrows); C) Coronal CT reconstruction demonstrating the extent of the hepatic collection, measuring approximately 8×7×6.2 cm (arrow).

An abdominopelvic computed tomography (CT) scan revealed a heterogeneous collection with confluent microcystic areas and irregular margins, measuring 8×7×6.2 cm (Figure 1).

While awaiting the results of the laboratory tests, a liver biopsy was performed primarily to rule out a neoplastic etiology, given the prolonged course of the clinical presentation and the lack of microbiological pathogen identification. Histopathological analysis revealed a chronic inflammatory process with a dense lymphoplasmacytic infiltrate, abundant eosinophils, and large granulomas with prominent necrosis.

Serological testing was positive for *Fasciola hepatica*, with a titer of 1:320 (reference value <1:160), and the diagnosis was confirmed by immunoblot. The patient was treated with triclabendazole 10 mg/kg/day in two divided doses, with marked clinical improvement.

Following treatment, laboratory parameters improved significantly, including normalization of eosinophil count (from 0.87 × 10^9^/L to 0.08 × 10^9^/L), inflammatory markers (C-reactive protein from 6.46 mg/dL to 0.08 mg/dL), and cholestatic liver enzymes (alkaline phosphatase from 236 U/L to 84 U/L and GGT from 168 U/L to 28 U/L), along with improvement in transaminases (ALT/AST from 114/95 U/L to 46/31 U/L) (Table 1).

**Figure 2:**
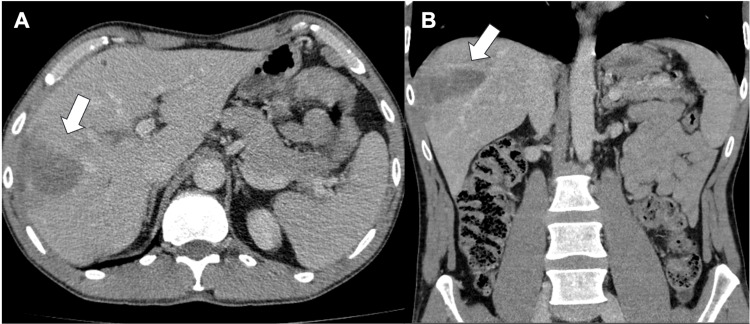
Abdominal CT scan showing the liver after treatment A) Axial abdominal computed tomography (CT) image showing a poorly defined, heterogeneous, hypodense area in the right mid-lobe of the liver (arrow); B) Coronal CT reconstruction showing the extent of the lesion, measuring approximately 3×6×6 cm, and appearing less organized compared to the previous examination.

At the three-month follow-up, imaging demonstrated a significant reduction in the size of the hepatic abscess and complete resolution of the portal vein thrombosis (Figure 2).

## Discussion

The increase in global mobility over the past decades has had a significant impact on the epidemiology of various infectious diseases, including fascioliasis. Although most cases of fascioliasis in Portugal are autochthonous [10], it is important to maintain heightened surveillance for imported cases, as illustrated by the patient described above, who had recently traveled to Cape Verde.

Clinical manifestations are highly variable and are divided into two phases: the acute phase, characterized by hepatic invasion and nonspecific symptoms; and the chronic phase, which is generally asymptomatic but may be associated with biliary complications [12].

Presentation with a hepatic abscess associated with PVT secondary to parasitic infection has been described in schistosomiasis and hydatid disease, but it is extremely rare in fascioliasis [13], and has not previously been reported in the national literature.

PVT can occur through various mechanisms, including the local compression of portal branches by the hepatic abscess, favoring stasis and thrombosis, or via hepatic-peritoneal larval migration, which induces granulomatous inflammation and hypereosinophilia with the release of procoagulant cytokines (Interleukin-5 (IL-5); Major Basic Protein (MBP)), contributing to a systemic procoagulant state [13].

Laboratory findings in these patients often include, in addition to elevated inflammatory markers, leukocytosis with eosinophilia during the acute phase, as well as altered liver function tests [12]. The absence of eosinophilia and abnormal liver tests during the initial phase made early diagnosis of this parasitic infection more difficult.

Diagnosis of fascioliasis is based on the identification of eggs in stool, duodenal aspirate, or bile, positive serology, and is complemented by imaging studies, including ultrasound, CT, or magnetic resonance imaging (MRI) [14]. Imaging findings can be nonspecific and make diagnosis more challenging in the absence of clinical suspicion. In general, both ultrasound and CT may appear normal, which is the most frequent scenario [4]. However, CT can reveal hypodense, subcapsular, tortuous lesions consistent with *Fasciola* migration tracts [4].

Liver biopsy is generally not required for diagnosis. Typical pathological findings include granulomatous inflammation with or without eggs, diffuse eosinophilic infiltration, migration tracts, Charcot-Leyden crystals, necrotic debris, and fibrosis [15].

Given the delay in obtaining serological tests, the prolonged clinical course, and the presence of segmental portal vein thrombosis, a liver biopsy was performed in our patient primarily to exclude neoplastic lesions, but also to rule out granulomatous disease or infestation by other hepatotropic parasites. Positive *Fasciola* serology confirmed by immunoblot allowed for definitive diagnosis, which was corroborated by histopathological examination of the liver biopsy.

Regarding treatment, triclabendazole is the drug of choice, as it is easy to administer, well tolerated, and has low rates of resistance. The recommended dose is 10 mg/kg/day, given as a single dose or divided into two doses 12 hours apart [11,16]. After targeted therapy, the patient showed excellent clinical response, with normalization of eosinophilia, reduction of the hepatic abscess and recanalization of the portal vein at three months of follow-up.

## Conclusions

This case underscores the importance of including fascioliasis in the differential diagnosis of hepatic abscesses, particularly in patients with relevant epidemiological risk factors and poor response to conventional antibiotic therapy. Although improvements in sanitation have reduced its incidence in Portugal, globalization and increased travel have contributed to imported cases, highlighting the need for continued clinical vigilance. Increased awareness and medical education regarding neglected tropical diseases are essential to ensure early diagnosis and appropriate treatment, preventing chronic disease and complications such as hepatic abscess formation and PVT.
